# Phytochemical and Biological Studies of *Nepeta asterotricha* Rech. f. (Lamiaceae): Isolation of Nepetamoside

**DOI:** 10.3390/molecules24091684

**Published:** 2019-04-30

**Authors:** Seyed Mostafa Goldansaz, Carmen Festa, Ester Pagano, Simona De Marino, Claudia Finamore, Olga Alessandra Parisi, Francesca Borrelli, Ali Sonboli, Maria Valeria D’Auria

**Affiliations:** 1 Department of Biology, Medicinal Plants and Drugs Research Institute, Shahid Beheshti University, G.C. Evin, 1983969411Tehran, Iran; mostafagoldansaz@gmail.com (S.M.G.); a-sonboli@sbu.ac.ir (A.S.); 2 Department of Pharmacy, School of Medicine and Surgery, University of Naples “Federico II”, Via D. Montesano 49, 80131 Naples, Italy; carmen.festa@unina.it (C.F.); ester.pagano@unina.it (E.P.); sidemari@unina.it (S.D.M.); claudia.finamorefinamore@unina.it (C.F.); olgaparisi7@gmail.com (O.A.P.); francesca.borrelli@unina.it (F.B.)

**Keywords:** *Nepeta asterotricha* Rech. f., Labiatae, secondary metabolites, iridoid glycosides, nepetamoside, NMR, anti-inflammatory effect, nitrites, cytokines

## Abstract

The *n*-butanolic extract, from an Iranian specimen of *Nepeta asterotricha* Rech. f. (NABE), displayed anti-inflammatory effects on lipopolysaccharide (LPS)-stimulated J774A.1 macrophages, which reduced nitrites and cytokines production. Bioassay guided fractionation of the extract led to the isolation of four iridoid glycosides, including a new one known as nepetamoside (**1**), one hexenyl-diglycoside, and some polyphenol and flavonoid components. None of the isolated iridoid components displayed significant effects on nitrites formation in an in vitro LPS-induced model of inflammation, thus suggesting that the plant anti-inflammatory effect is probably due to a synergistic action among its constituents.

## 1. Introduction

*Nepeta* L. (tribe Mentheae: subtribe Nepetoideae), is one of the largest genera in the Lamiaceae family. It represents more than 280 species, displaying a significant diversity in growth forms, pollination biology, floral morphology, and secondary metabolites [1] *Nepeta* is represented in the flora of Iran by 79 species, 42 of which, such as *Nepeta asterotricha* Rech. f., are endemic [2,3].

Decoctions and infusions from the aerial parts of *Nepeta* species have been used in traditional and folk medicines as antioxidant, anticonvulsant, tonic, antipyretic, antitussive, sedative, antiasthmatic, antispasmodic and anti-inflammatory medicines [4,5]. Some of these therapeutic actions were recently comprehensively reviewed [6] and received a modern pharmacological validation [7,8,9].

So far, the phytochemical study of the extracts from *Nepeta* species has mainly been devoted to the analysis of the volatile constituents in the essential oil [10,11]. Although the chemical composition of the volatile oils has been found to be influenced by external factors such as climatic and ecological conditions, plant organ and vegetative cycle stage, there were roughly two different chemotypes of *Nepeta* spp., the first one containing nepetalactone diastereoisomers as main constituents, whereas the second one contains 1,8-cineole (eucaliptol) as the predominant component. The chemical composition of the essential oils from root, leaf and aerial part of *Nepeta asterotricha* Rech. f. has been investigated and 1,8-cineole and nepetalactone have been identified as the major oil components of the leaf/aerial and root, respectively [12]. Moreover, biological studies revealed that the essential oil possesses antimicrobial and antifungal activities.

In this paper, we report the phytochemical study of the methanol extract from *Nepeta asterotricha* Rech. f. from Iran. Moreover, in order to validate the plant use in folk medicine, the anti-inflammatory potential of the *Nepeta asterotricha* extract as well as of the enriched fractions and isolated compounds was also investigated.

Bioassay guided fractionation of the crude methanol extract led to the isolation of four iridoid components, including a new one which we named nepetamoside, and to the identification of several known polyphenols and flavonoids components.

In vitro studies on LPS-stimulated macrophages revealed that the methanol extract of *Nepeta asterotricha* possessed anti-inflammatory effects by reducing the formation of nitrites and cytokines induced by lipopolysaccharide (LPS) in macrophages.

## 2. Results

### Phytochemical Investigation and Evaluation of Biological Activity

Air-dried leaves of *Nepeta asterotricha* were ground and extracted with MeOH. Removal of the solvent under reduced pressure yielded a crude extract which was subjected to solvent partitioning using a Kupchan’s modified procedure to obtain four different fractions (*n*-hexane, CCl_4_, CHCl_3_ and *n*-BuOH). Among the fractions, the *Nepeta asterotricha n*-BuOH extract (NABE, 9.2 g) showed anti-inflammatory activity in the lipopolysaccharide (LPS)-stimulated J774A.1 macrophages.

**Figure 1:**
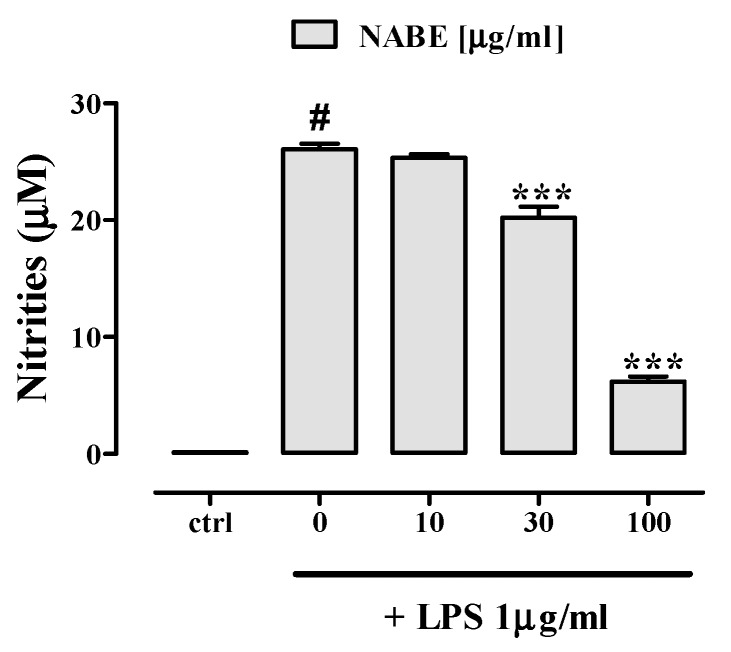
Inhibitory effect of *Nepeta asterotricha n-*butanol extract (NABE) on nitrite levels in the cell medium of J774A.1 macrophages stimulated with lipopolysaccharide (LPS, 1 μg/mL) for 24 h. NABE, at the concentration range of 10–100 μg/mL, were added to the cell media 30 min before LPS stimulus. Results are expressed as mean ± SEM of three experiments (in triplicates). # *p* < 0.001 vs control (ctrl); *** *p* < 0.001 vs. LPS alone.

**Figure 2:**
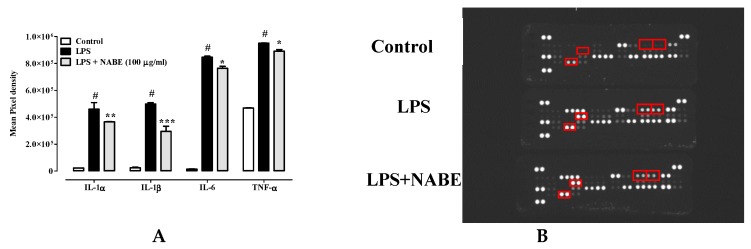
(**A**) Inhibitory effect of *Nepeta asterotricha n-*butanolic extract (NABE) on inflammatory cytokines levels [i.e., IL-1α, IL-1β, IL-6 and TNF-α] in the cell medium of J774A.1 macrophages stimulated with lipopolysaccharide (LPS, 1 μg/mL) for 24 h. NABE, used at the concentration of 100 μg/mL, were added to the cell media 30 min before LPS stimulus. (**B**) Representative images of each array (untreated, treated with LPS and LPS+ NABE). Results are expressed as mean ± SEM of three experiments (in triplicates). # *p* < 0.001 vs control; * *p* < 0.05, ** *p* < 0.01 and *** *p* < 0.001 vs LPS alone.

Macrophages, a heterogenous population of innate immune cells, are the key actors in the inflammation pathogenesis, by playing a crucial role in host defence and by controlling tissue-specific signals [13]. Their activation, mainly by LPS, is associated with the transcription of genes that encode for the production of inflammation-related mediators such as nitric oxide and cytokines [e.g., tumor necrosis factor-α (TNF-α), interleukin-6 (IL-6), interleukin-1β (IL-1β)] [14,15]. Therefore, we investigated the effect of NABE, its fractions and isolated compounds in J774A.1 macrophages stimulated by LPS. Treatment of J774A.1 macrophages with the inflammatory agent LPS (1 μg/mL for 24 h) caused a significant increase in nitrite (the stable form of nitric oxide) levels which was significantly reduced, in a concentration-dependent manner, by a pre-treatment with NABE at the concentration of 10–100 μg/mL, 30 min before LPS addition (Figure 1). Modulation of NO synthesis represents an important approach for the treatment of inflammatory conditions [16]. Of note, in macrophages not treated with LPS, NABE did not modify, *per se*, basal nitrite levels (data not shown). NABE, at concentrations ranging from 10 to 100 μg/mL, did not affect the cell vitality after 24 h of exposure, thus suggesting an absence of cytotoxic effects at these concentrations. (Control 100 ± 2.5; NABE 10 µg/mL 99.8 ± 3.2; NABE 30 µg/mL 99.6 ± 3.3; NABE 100 µg/mL 98.9 ± 3.8. Mean ± SEM of three independent experiments, each performed in octuplicate). By contrast, NABE at a concentration of 300 µg/mL induced a significant inhibition of macrophages viability (Control 100 ± 2.5; NABE 300 µg/mL 86.8 ± 2.3; *p* < 0.05. Mean ± SEM of three independent experiments, each performed in octuplicate). DMSO 20% was used as a positive control and significantly reduced cell viability (Control 100 ± 2.5; DMSO 20% 20.8 ± 1.2; *** *p* < 0.001. Mean ± SEM of three independent experiments, each performed in octuplicate). The anti-inflammatory effect of NABE was corroborated by quantification of a cytokines panel as assessed by a Proteome Profiler Mouse Cytokine Array Kit that is a membrane-based sandwich immunoassay. By using this assay, we measured, at the same time, the levels of 40 pro-inflammatory and anti-inflammatory secreted cytokine proteins in LPS-stimulated J774. According to literature [14], LPS (1 µg/mL) determined a significant increase of several cytokines, including interleukin (IL)-1α, IL-1β, IL-6 and TNF-α in macrophages (Figure 2). Among the screened cytokines, NABE (100 μg/mL) significantly decreased the production of these key pro-inflammatory cytokines in macrophages stimulated by LPS (Figure 2). However, NABE (100 μg/mL) did not affect the production of anti-inflammatory cytokines (e.g., IL-10) (data not shown).

In order to identify the substance/s responsible for the NABE anti-inflammatory effect, an aliquot of NABE (2.4 g) was fractionated by DCCC to afford 12 fractions. Among the fractions, fractions 5, 6, 7, 11 and 12 (100 µg/mL) were able to significantly reduce the formation of nitrites induced by LPS (Control 5.6 ± 0.06; LPS 25.9 ± 0.31^#^; fraction 5 22.8 ± 0.32 *; fraction 6 21.3 ± 0.93 **; fraction 7 21.8 ± 0.94 **; fraction 11 18.5 ± 0.18 ***; fraction 12 7.98 ± 0.28 ***, ^#^ *p* < 0.001 vs. control, * *p* < 0.05, ** *p* < 0.01 and *** *p* < 0.001 vs. LPS, *n* = 3) being most effective on fraction 12 (% of inhibition: 88.3 %). All these fractions at the concentration of 100 µg/mL did not affect cell viability (data not shown).

**Figure 3:**
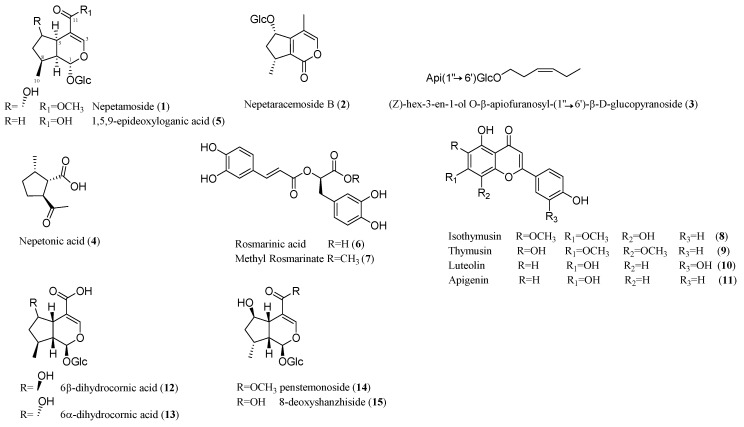
New (**1**) and known (**2**–**11**) compounds isolated from *Nepeta asterotricha* Rech. f. and compounds **12**–**15** as reference compounds reported in Table 1 and Table 2.

However, for a complete phytochemical investigation, all fractions were subjected to further purification (see Materials and Methods for details) by reversed phase HPLC to afford a new iridoid glucoside (**1**) together with know nepetaracemoside B (**2**) [17], (*Z*)-hex-3-en-1-ol *O*-β-apiofuranosyl-(1′′→6′)-β-d-glucopyranoside (**3**) [18] nepetonic acid (**4**) [19], and 1,5,9-*epi*-deoxyloganic acid glucosyl ester (**5**) [20] (Figure 3).

Compound **1** was isolated as a colorless oil. C_17_H_26_O_10_ molecular formula was deduced from the sodiated molecular ion peak at *m*/*z* 413.1411 (calcd. 413.1418) observed in the HRESIMS, together with the ^13^C-NMR spectroscopic data (see Supplementary Materials). The ^1^H spectrum (Table 1) displayed the diagnostic carbinol proton signals relative to a β-glucopyranose unit, including the anomeric proton signal at δ_H_ 4.56 (1H, d, *J* = 7.9 Hz), and the signals relative to the diasteroisomeric methylene protons at C-6′: 3.84 (1H, d, *J* = 11.8 Hz) and 3.68 (1H, dd, *J* = 4.0, 11.8 Hz). The ^13^C-NMR chemical shifts, with one anomeric carbon at δ_C_ 104.2, four methine signals between 71.2 and 78.3 ppm and one methylene at δ_C_ 62.5 confirmed the presence of the above monosaccharide unit.

**Figure 4:**
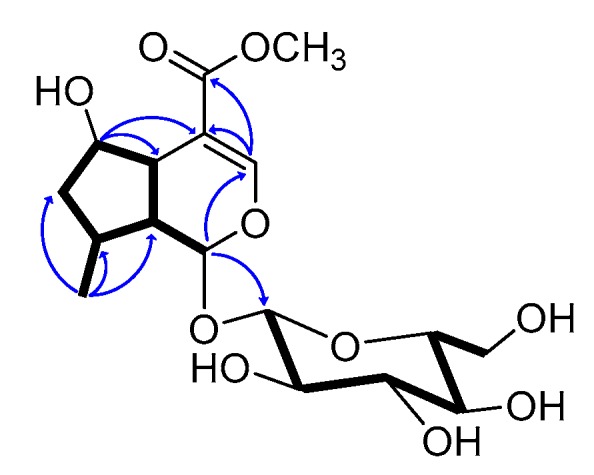
COSY connectivities (bold bonds) and key HMBC correlations (blue arrows) for nepetamoside (**1**).

Besides the sugar unit, ^13^C-NMR spectrum also showed eleven carbons, one methyl (δ_C_ 16.7), three sp^3^ methines (δ_C_ 33.5, 42.1 and 42.7), one sp^3^ methylene (δ_C_ 41.9), one α,β-unsaturated methyl ester group (δ_C_ 169.5, 154.4, 110.9, 51.8), one ketal carbon (δ_C_ 100.7), and one carbinol secondary carbon (δ_C_ 77.7) that were reminiscent of an iridoid aglycone [21,22,23]. The analysis of ^1^H-^1^H COSY allowed us to assign the spin system C-1-C-9/C-5 (bold in Figure 4) and to place the hydroxyl group at C-6, on the basis of the chemical shift of the H-6 proton (δ_H_ 4.19) and the methyl group at C-8 (δ_H_ 2.58). The functionalization of the pyrane ring as well as the glycosidation at C-1 were determined by the diagnostic HMBC correlations in Figure 4. The assigned planar structure was found to be identical to that of penstemonoside, originally [24] isolated from *Penstemon barbatus*, and then reported from *Castilleja rhexifolia* [25].

As reported in the literature [26], due to the high flexibility of the iridoid skeleton, the dipolar couplings cannot be used as parameters for the configurational assignment. We assign a *cis*-fused ring structure [27] on the basis of the ^1^H and ^13^C NMR resonances of C-1, C-5 and C-9, together with the relative scalar coupling constants. The careful comparison of the NMR spectral data (Table 1 and Table 2) of 6β-dihydrocornic acid (**12**) and 6α-dihydrocornic acid (**13**) [28], penstemonoside (**14**) [24], 8-dihydroshanzhiside (**15**) [29], (Figure 3), indicated a relative disposition of the hydroxyl and methyl groups at C-6 and C-8 respectively as in penstemonoside and 8-dihydroshanzhiside, the stereostructure of which has been secured by X-ray investigation.

However, the ^13^C-NMR data of compound **1**, albeit similar to that of penstemonoside, were not superimposable. The most significant difference is related to C-1 and C-1′ chemical shifts.

By analogy with previous literature data [30], we envisioned an enantiomeric configuration in the aglycone portion of **1**, with respect to penstemonoside. This presumption was confirmed by consistent evidence. First, the specific rotation values of the iridoid glycosides with “classic” (1*R*, 5*R*, 9*S*) stereostructure were reported as invariably negative, whereas the iridoid glycosides from plants of *Nepeta* genus with a distinctive enantiomeric (1*S*, 5*S*, 9*R*) stereostructure displayed positive optical rotation values. The positive value of specific rotation of compound **1** was in agreement with a (1*S*,5*S*,9*R*) stereostructure. Analogously, the CD spectrum of **1** displayed a positive Cotton effect, as reported for iridoids from *Nepeta* [31]. Moreover, the enzymatic hydrolysis of **1** led to a C-1 epimeric mixture of aglycones, whose ^1^H-NMR data were superimposable to those reported for the aglycone of penstemonoside [25].

Therefore, compound **1** was determined to be *ent*-1,5,6,8,9-penstemonoside for which we propose the trivial name nepetamoside.

The fraction 12 from DCCC, the more active fraction in the nitrite assay, containing a complex mixture of flavonoids and polyphenol components, was first further purified by Sephadex^®^ LH-20 and then by reversed phase HPLC to afford rosmarinic acid (**6**), methyl rosmarinate (**7**), 8-hydroxycirsimaritin (also named isothymusin) (**8**), thymusin (**9**), luteolin (**10**) and apigenin (**11**) (Figure 3). The structure of the known compounds was elucidated by comparison of the spectral data with those reported in the literature.

Several studies reported the determination of flavonoids and phenolic acids profile [32] of aerial parts of *Nepeta* species although in most cases the identification of the individual components was mainly performed by HPLC analysis. In particular, a comprehensive study on 38 species of *Nepeta* [33], including *Nepeta asterotricha*, analyzed some common features in the composition of flavonoids. Our analysis gave results similar to those reported for the same species, with 8-hydroxycirsimaritin (**8**), which represents a chemotaxonomic marker of the *Nepeta* genus as a major flavonoid component.

Our analysis didn’t reveal the presence of genkwanin and cirsimaritin usually reported as additional major components of this fraction.

Concerning the phenolic acid components, whereas rosmarinic acid (**6**) was reported as a major component in some *Nepeta* species [30], its methyl ester (**7**) has so far been isolated only from a Chinese specimen of *Nepeta pratii* [34].

All the phenolic compounds isolated in the present study are very common chemical constituents of several plants and, as single components, have been subjected to extensive pharmacological investigation for their anti-inflammatory potential. Only 8-hydroxycirsimaritin (**8**) has not been pharmacologically characterized. In our screening 8-hydroxycismaritin didn’t affect the nitrite production in LPS-stimulated J774A.1 macrophages. However, since 8-hydroxycirsimaritin is a high chemically unstable compound [35], we could assume that the observed inactivity may be caused by chemical decomposition during acquisition of the data.

The anti-inflammatory effects of rosmarinic acid (**6**) and apigenin (**11**) (the main components found in NABE) have been extensively demonstrated [36,37,38]. Therefore, it has been presumed that the anti-inflammatory effect of NABE could be due to the presence of these compounds. However, the amount of these compounds found in NABE is too low to exert anti-inflammatory effects, therefore, it is believed that the overall effect of NABE on nitrites and cytokines productions is probably due to a synergistic action of all components (found in NABE) rather than due to a single compound.

## 3. Materials and Methods

### 3.1. General Information

Specific rotations were measured on a Perkin Elmer 243 B polarimeter (Waltham, MA, USA). Micromass Q-TOF mass spectrometer was used to perform high-resolution ESI-MS spectra (Q-TOF premier, Waters Co., Milford, MA, USA).

NMR spectra were recorded on Varian Inova 400 and 500 NMR spectrometers (Palo Alto, CA, USA) (^1^H at 400 and ^13^C at 100, ^1^H at 500 MHz and ^13^C at 125 MHz, respectively), equipped with a SUN microsystem ultra 5 hardware. Coupling constants (*J* values) are reported in Hertz (Hz), and chemical shifts (δ) in ppm, referred to CHD_2_OD (δ_H_ 3.31 and δ_C_ 49.0). Spin multiplicities are given as s (singlet), br s (broad singlet), d (doublet), t (triplet) or m (multiplet). Through-space ^1^H connectivities were obtained using a ROESY experiment with mixing times of 150, 200 and 250 ms.

DCCC was performed using a DCC-A (Rakakikai Co. Di Tokio, Japan) equipped with 250 columns (internal diameter 3 mm). Silica gel (200–400 mesh) from Macherey-Nagel Company and Sephadex^®^ LH-20 from Sigma Aldrich (St. Louis, MO, USA) was used for chromatography.

HPLC was performed using a Waters Model 510 pump equipped with Waters Rheodine injector and a differential refractometer, model 401 experiment (Waters Co., Milford, MA, USA).

### 3.2. Plant Material

The aerial parts of Nepeta asterotricha were collected at full flowering time in Deh-Bala, Yazd, Iran. The sample were identified and placed in Herbarium of Medicinal Plants and Drugs Research Institute, Shahid Beheshti University, Tehran, Iran. The sample code in the Herbarium is MPH-2569.

### 3.3. Extraction and Isolation

The aerial parts of the plant (230 g) were extracted with methanol (3 × 1 L) at room temperature and the crude methanolic extract was subjected to a modified Kupchan’s partitioning procedure as follows. The methanol extract was dissolved in a mixture of MeOH/H_2_O containing 10% H_2_O and partitioned against n-hexane (3.5 g). The water content (% *v*/*v*) of the MeOH extract was adjusted to 30% and partitioned against CHCl_3_ (18.7 g). The aqueous phase was concentrated to remove MeOH and then extracted with n-BuOH (9.2 g). An aliquot of n-BuOH extract (2.4 g) was submitted to droplet counter-current chromatography (DCCC) with n-BuOH/Me_2_CO/H_2_O (3:1:5) in descending mode (the upper phase was the stationary phase). The obtained fractions were monitored by TLC on Silica gel plates with n-BuOH/AcOH/H_2_O (12:3:5) and CHCl_3_/MeOH/H_2_O (80:18:2) as eluents. Twelve fractions were obtained and purified by HPLC on a Nucleodur 100-5 C18 column (5 μm, 4.6 mm i.d × 250 mm) using MeOH/H_2_O (4:6) as eluent. An aliquot of fraction 7 (50 mg), containing exclusively nepetamoside (**1**), was purified in HPLC to give 17 mg of pure compound **1** (t_R_ = 14.2 min). Fraction 9 (30.1 mg) afforded 3.8 mg of nepetaracemoside B (**2**) (t_R_ = 6.5 min) and 4.2 mg of (Z)-hex-3-en-1-ol *O*-β-apiofuranosyl-(1″→6′)-β-d-glucopyranoside (**3**) (t_R_ = 26 min), fraction 10 (270 mg) contained exclusively nepetonic acid (**4**) (62 mg; t_R_ = 30 min) and fraction 11 (548 mg) afforded 23.4 mg of 1,5,9-epi-deoxyloganic acid (**5**) (t_R_ = 24 min).

Fraction 12 was further chromatographed on Sephadex^®^ LH-20 (MeOH eluent). Fractions were collected and combined according to TLC analysis into nine main fractions (A–I). Fraction G (37.6 mg) was further purified by HPLC with MeOH/H_2_O 1:1 (flow rate 1.0 mL/min) to give 3.6 mg of rosmarinic acid (**6**) (t_R_ = 3.5 min), 1.0 mg of methyl rosmarinate (**7**) (t_R_ = 12 min), 9.4 mg of 8-hydroxycirsimaritin (**8**) (t_R_ = 31.5 min), 0.7 mg of thymusin (**9**) (t_R_ = 50 min). Fraction H (7.7 mg) was separated by HPLC with MeOH/H_2_O 1:1 (flow rate 1.0 mL/min) obtaining 1.1 mg of luteolin (**10**) (t_R_ = 28.5 min) and 0.8 mg of apigenin (**11**) (t_R_ = 48.4 min).

#### 3.3.1. Nepetamoside (**1**)

Yellow amorphous solid; [α${]}_{D}^{25}$ + 81.4 (c 0.60, MeOH); ^1^H and ^13^C-NMR data in CD_3_OD given in Table 1 and Table 2; HRESIMS (positive-ion mode) m/z 413.1411 [M + Na]^+^ (calcd. for C_17_H_26_O_10_Na, 413.1418).

#### 3.3.2. Nepetaracemoside B (**2**)

Yellow amorphous solid; [α${]}_{D}^{25}$ − 4.0 (c 0.12, MeOH); ^1^H and ^13^C-NMR data were identical to those previously reported in literature [17]; HRESIMS (positive-ion mode) m/z 365.1212 [M + Na]^+^ (calcd. for C_16_H_22_O_8_Na, 365.1207).

#### 3.3.3. (Z)-hex-3-en-1-ol *O*-β-apiofuranosyl-(1″→6′)-β-d-glucopyranoside (**3**)

White amorphous solid; ^1^H and ^13^C-NMR data were identical to those previously reported in literature [18]. HRESIMS (positive-ion mode) m/z 417.1729 [M + Na]^+^ (calcd. for C_17_H_30_O_10_Na, 417.1731).

#### 3.3.4. Nepetonic Acid (**4**)

Yellow amorphous solid; [α${]}_{D}^{25}$ + 16.6 (c 0.79, MeOH); ^1^H and ^13^C-NMR data were identical to those previously reported in literature [19]; HRESIMS (negative-ion mode) *m*/*z* 169.0869 [M − H]^−^ (calcd. for C_9_H_13_O_3_, 169.0870).

#### 3.3.5. 1.5.9-epi-deoxyloganic Acid (**5**)

Yellow amorphous solid; [α${]}_{D}^{25}$ + 65.7 (c 1.61, MeOH); ^1^H and ^13^C-NMR data were identical to those previously reported in literature [20]; HRESIMS (negative-ion mode) m/z 359.1345 [M − H]^−^ (calcd. for C_16_H_23_O_9_, 359.1348).

#### 3.3.6. Rosmarinic Acid (**6**)

Yellow amorphous solid; ^1^H and ^13^C-NMR data were identical to those previously reported in literature [39,40]. HRESIMS (negative-ion mode) *m*/*z* 359.0773 [M − H]^−^ (calcd. for C_18_H_15_O_8_, 359.0779).

#### 3.3.7. Methyl Rosmarinate (**7**)

Yellow amorphous solid; Spectroscopic data were in agreement with those reported in literature [40]; HRESIMS (positive-ion mode) *m*/*z* 397.0902 [M + Na]^+^ (calcd. for C_19_H_18_O_8_Na, 397.0894).

#### 3.3.8. 8-Hydroxycirsimaritin or Isothymusin (**8**)

Yellow amorphous solid; Spectroscopic data were in agreement with those reported in literature [41]; HRESIMS (positive-ion mode) m/z 353.0639 [M + Na]^+^ (calcd. for C_17_H_14_O_7_Na, 353.0632).

#### 3.3.9. Thymusin (**9**)

Yellow amorphous solid; Spectroscopic data were in agreement with those reported in literature [41]; HRESIMS (positive-ion mode) *m*/*z* 353.0637 [M + Na]^+^ (calcd. for C_17_H_14_O_7_Na, 353.0632).

#### 3.3.10. Luteolin (**10**)

Yellow amorphous solid; ^1^H and ^13^C-NMR data were identical to those previously reported in literature [42]; HRESIMS (positive-ion mode) *m*/*z* 309.0372 [M + Na]^+^ (calcd. for C_15_H_10_O_6_Na, 309.0370).

#### 3.3.11. Apigenin (**11**)

Yellow powder;^1^H and ^13^C-NMR data were identical to those previously reported in literature [42]; HRESIMS (positive-ion mode) *m*/*z* 293.0426 [M + Na]^+^ (calcd. for C_15_H_10_O_5_Na, 293.0420).

### 3.4. Enzymatic Hydrolysis of Nepetamoside 1

A solution of **1** (2 mg) in H_2_O (1 mL) was hydrolyzed with β-glucosidase at 37 °C for 24 h. After reaction for 12 h, the TLC analysis (SiO_2_ with n-BuOH-AcOH-H_2_O 60:15:25) showed that the starting material has disappeared. The mixture was passed through a C-18 SEP-PACK cartridge, washed with H_2_O and eluted with MeOH. The MeOH eluate gave the aglycone epimeric mixture. Selected ^1^H-NMR data (400 MHz, CD_3_OD) of 1α and 1β epimers: δ_H_ 1.10 (d, *J* = 7.0 Hz, H_3_-10), 3.77 (s, OCH_3_), 4.07/4.24 (br singlets, H-6), 4.98/5.42 (br singlets H-l) and 7.44 (s, H-3). HRESIMS *m*/*z* 251.0893 [M + Na]^+^ (calcd. for C_11_H_16_O_5_Na, 251.0890).

### 3.5. Biological Assays

#### 3.5.1. Cell Culture

J774A.1 macrophages (ATCC, from LGC Standards, Milan, Italy) were used for in vitro experiments. Macrophages were routinely maintained at 37 °C in a humidified atmosphere of 5% CO_2_ and were cultured in Dulbecco’s Modified Eagle’s medium (DMEM, Lonza Group, Milan, Italy) supplemented with 10% fetal bovine serum (FBS, Sigma Aldrich, Milan, Italy) 100 U/mL penicillin and 100 μg/L streptomycin, 2 mM l-glutamine, 20 mM Hepes [4-(2-hydroxyethyl)-1-piperazineethanesulphonic acid] and 1 mM Na pyruvate. The medium was changed every 48 h in conformity with the manufacturer’s protocols.

#### 3.5.2. Cell Viability

Cell viability was evaluated by measuring the incorporation of neutral red (NR), a weak cationic dye, in lysosomes (NR assay). Briefly, J774A.1 macrophages were seeded in 96-well plates at a density of 3 × 10^4^ cells per well and were allowed to adhere for 24 h. Then, cells were treated with the Nepeta asterotricha n-butanolic extract (NABE, 10–100 µg/mL) for 24 h. Subsequently, macrophages were incubated with NR dye solution (50 μg/mL in medium) for 3 h at 37 °C and then lysed with 1% acetic acid. The absorbance was read at 532 nm (iMarkTM microplate reader, Bio-Rad, Milan, Italy). All experiments were performed in triplicate (including 8 replicates for each treatment).

#### 3.5.3. Nitrite Measurement and Pharmacological Treatment In Vitro

The effect of NABE on the nitric oxide (NO) production was assessed by measuring nitrites, stable metabolites of NO, in macrophages medium via a colorimetric assay, as previously described [43]. J774A.1 macrophages (5 × 10^5^ cells per well seeded in a 24-well plate) were incubated with NABE (10–100 µg/mL) for 30 min and subsequently with LPS (1 μg/mL) for 24 h. Then, the cell supernatant was collected and incubated with 100 µL of Griess reagent (0.2% naphthylethylenediamine dihydrochloride and 2% sulphanilamide in 5% phosphoric acid) at room temperature for 10 min in order to allowed the formation of a colored azo dye. The absorbance was read at 550 nm. Serial-diluted sodium nitrite (Sigma-Aldrich, Milan, Italy) was used to generate a standard curve. The data were expressed as µM of nitrite. Each sample was determined in triplicate.

#### 3.5.4. Protein Array Analysis

In order to confirm the anti-inflammatory effect of NABE, we also measured the levels of proinflammatory and antinflammatory secreted cytokine proteins in LPS-stimulated macrophages by using the Proteome Profiler Mouse Cytokine Array Kit, Panel A (R&D system, Space import export Srl, Milan, Italy) following the manufacturer’s instructions. Briefly, J774A.1 macrophages were incubated with NABE at concentration of 100 µg/mL for 30 min and subsequently with LPS (1 μg/mL) for 24 h. Successively, cell supernatants were incubated with Mouse Cytokine Detection Antibody Cocktail for 1 h at room temperature. Then, the samples were incubated with a membrane containing 40 different anti-cytokine antibodies printed in duplicate at 4 °C overnight on a rocking platform shaker. The unbound proteins were removed and the membranes were washed three times with a washing buffer. The Streptavidin-HRP solution was then added to the membranes on a rocking platform shaker for 30 min. After being washed three times, the protein spots were visualized using the chemiluminescence detection reagents supplied in the Array Kits (R&D system, Space import export Srl, Milan, Italy). The intensity score of each duplicated array spot was measured with software ImageQuant Capture (GE Healthcare, Milan, Italy) and analyzed using Quantity One Software version 4.6.3 (Bio-Rad, Milan, Italy).

#### 3.5.5. Statistical Analysis

Results are expressed as the mean ± standard error of the mean (S.E.M.) of 3 independent experiments. To determine statistical significance, a Student’s t-test was used for comparing a single treatment mean with a control mean, and a one-way analysis of variance followed by a Tukey–Kramer multiple comparisons test was used for analysis of multiple treatment means. A value of *p* < 0.05 was considered significant.

## 4. Conclusions

To the best of our knowledge, this is the first paper that has both analyzed the chemical composition of the aerial parts of *Nepeta asterotricha* and evaluated its anti-inflammatory action.

Phytochemical analysis revealed the presence of several already known iridoids, a new iridoid glycoside and several known polyphenols and flavonoids. As a result of the use of macrophages, we also demonstrated that the *n*-butanolic extract of *Nepeta asterotricha* exerted anti-inflammatory effects which are probably due to a synergistic action among the components present in the extract.

In conclusion, our results validate the traditional use of *Nepeta asterotricha* for the treatment of inflammatory conditions. Further pre-clinical studies should be performed in vivo to confirm the *Nepeta asterotricha* anti-inflammatory effect, as well as to assess its safety.

## Figures and Tables

**Table 1:** ^1^H-NMR data of Nepetamoside ^a^ (**1**), 6β-dihydrocornic acid (**12**) ^b^, 6α-dihydrocornic acid (**13**) ^b^, penstemonoside (**14**) ^c^ and 8-deoxyshanzhiside (**15**) ^c^.

C	1	12	13	14	15
1	5.39 br s	5.25 d (5)	5.21 d (9)	5.58 d (2.5)	5.44 d (2)
3	7.41 s	7.41 s	7.62 s	7.48 d (0.9)	7.34 s
4	-	-	-	-	-
5	2.85 t (8.5)	2.79 t (6)	2.82 dd (4, 9)	2.88 br d	2.70 d (9)
6	4.19 br s	4.05 m	4.47 t (4)	4.23 m	4.11 t (2)
7	1.40 ddd (4.6, 10.2, 13.7) 1.76 dd (7, 13.1)	1.25 m 2.17 m	1.38 ddd (4, 10, 13) 1.92 dd (8, 13)	1.80 m 1.50 ddd (4.2, 9.8, 14)	1.38 ddd (4, 10, 13) 1.65 dd (8, 13)
8	2.58 m	1.96 q (7)	2.30 m	2.58 m	2.43 m
9	2.80 m	2.03 dt (5, 6, 7)	1.70 dt (4, 8)	2.71 td (2.5, 9.3, 11.7)	2.56 dt (2, 9.2, 9,2)
10	1.05 d (7.2)	1.15 d (7)	1.12 d (8)	1.02 d (7.2)	0.87 d (7)
11	-	-	-	-	-
OMe	3.71 s	-	-	3.75 s	-
*Glc*					
1′	4.56 d (7.9)	4.65 d (8)	4.70 d (8)	4.76 d (8.1)	4.63 d (8)
2′	3.20 t (8.2)	3.20 t (8)	3.24 dd (8, 9)	3.25 dd (8.1, 9.3)	3.10 t (9)
3′	3.35 ovl	3.37 m	3.40 t (9)	3.30-3.51 m	3.33 t (9)
4′	3.31 ovl	3.37 m	3.31 m	3.30-3.51 m	3.23 t (9)
5′	3.29 ovl	3.30 m	3.29 m	3.30-3.51 m	3.33 t (9)
6′	3.68 dd (4, 11.8) 3.84 d (11.8)	3.67 dd (6, 12) 3.89 dd (2, 12)	3.67 dd (6, 12) 3.86 dd (2, 12)	3.72 dd (5.7, 12.3) 3.92 dd (2, 12.3)	3.62 dd (6, 12) 3.77 d (12)

^a^ Acquired in CD_3_OD at 400 MHz; Coupling constants (*J* in Hz) in parentheses; ^1^H and ^13^C assignments aided by ^1^H-^1^H COSY, HSQC, and HMBC experiments. ovl: overlapped with other signals. ^b^ In CD_3_OD as previously reported [28]. ^c^ In D_2_O as previously reported [25,28].

**Table 2:** ^13^C-NMR data of Nepetamoside ^a^ (**1**), 6β-dihydrocornic acid (**12**) ^b^, 6α-dihydrocornic acid (**13**) ^b^, penstemonoside (**14**) ^b^ and 8-deoxyshanzhiside (**15**) ^c.^

C	1	12	13	14	15
1	100.7	97.5	101.2	96.1	96.0
3	154.4	153.6	155.9	153.7	153.3
4	110.9	110.8	107.4	111.0	110.3
5	42.7	43.7	43.5	43.0	41.1
6	77.7	78.8	75.1	77.8	77.1
7	41.9	42.7	43.2	41.7	40.8
8	33.5	34.3	35.2	33.8	32.5
9	42.1	47.9	47.0	42.5	40.5
10	16.7	21.1	21.9	16.6	15.7
11	169.5	171.0	171.1	169.5	171.1
OMe	51.8	-	-	51.8	-
*Glc*					
1′	104.2	100.2	100.4	99.7	98.6
2′	75.2	74.8	74.9	74.5	72.9
3′	78.0	78.1	78.1	78.1	75.9
4′	71.2	71.7	71.7	71.5	69.9
5′	78.3	78.4	78.5	77.8	76.5
6′	62.5	62.8	63.0	62.7	61.0

^a^ Acquired in CD_3_OD at 400 MHz ^b^ In CD_3_OD as previously reported [28]. ^c^ In D_2_O as previously reported [25,28].

## Supplementary Materials

The following are available online. Figures S1–S6: ^1^H, ^13^C-NMR, COSY, HSQC, HMBC and HRESIMS spectra of compound **1**; Figures S7–S16: ^1^H-NMR spectra of compounds **2**–**11**.
