# Supplementary material for: Phytochemical and Biological Studies of Nepeta asterotricha Rech. f. (Lamiaceae): Isolation of Nepetamoside
Source: Molecules. 2019 Apr 30;24(9):1684. doi: 10.3390/molecules24091684 (PMC6539229; doi:10.3390/molecules24091684)

Supplementary Material

Phytochemical and Biological Studies of *Nepeta asterotricha* Rech. f. (Lamiaceae): Isolation of Nepetamoside

Seyed Mostafa Goldansaz[1,2], Carmen Festa[2], Ester Pagano[2], Simona De Marino[2], Claudia Finamore[2], Olga Alessandra Parisi[2], Francesca Borrelli[2], Ali Sonboli[1], Maria Valeria D'Auria[2,*]

1 Department of Biology, Medicinal Plants and Drugs Research Institute, Shahid Beheshti University, G.C. Evin, Tehran, Iran
2 Department of Pharmacy, School of Medicine and Surgery, University of Naples "Federico II", Via D. Montesano 49, 80131 Naples, Italy

Table of contents:

Figures S1 and S2. ^{1}H and ^{13}C NMR spectra of compound **1** S2
Figures S3 and S4. COSY and HSQC spectra of compound **1** S3
Figures S5 and S6. HMBC and HRESIMS spectra of compound **1** S4
Figures S7 and S8. ^{1}H-NMR spectra of compounds **2** and **3** S5
Figures S9 and S10. ^{1}H-NMR spectra of compounds **4** and **5** S6
Figures S11 and S12. ^{1}H-NMR spectra of compounds **6** and **7** S7
Figures S13 and S14. ^{1}H-NMR spectra of compounds **8** and **9** S8
Figures S15 and S16. ^{1}H-NMR spectra of compounds **10** and **11** S9

Figure S1. ^{1}H NMR (500 MHz, CD_3OD) of compound **1**

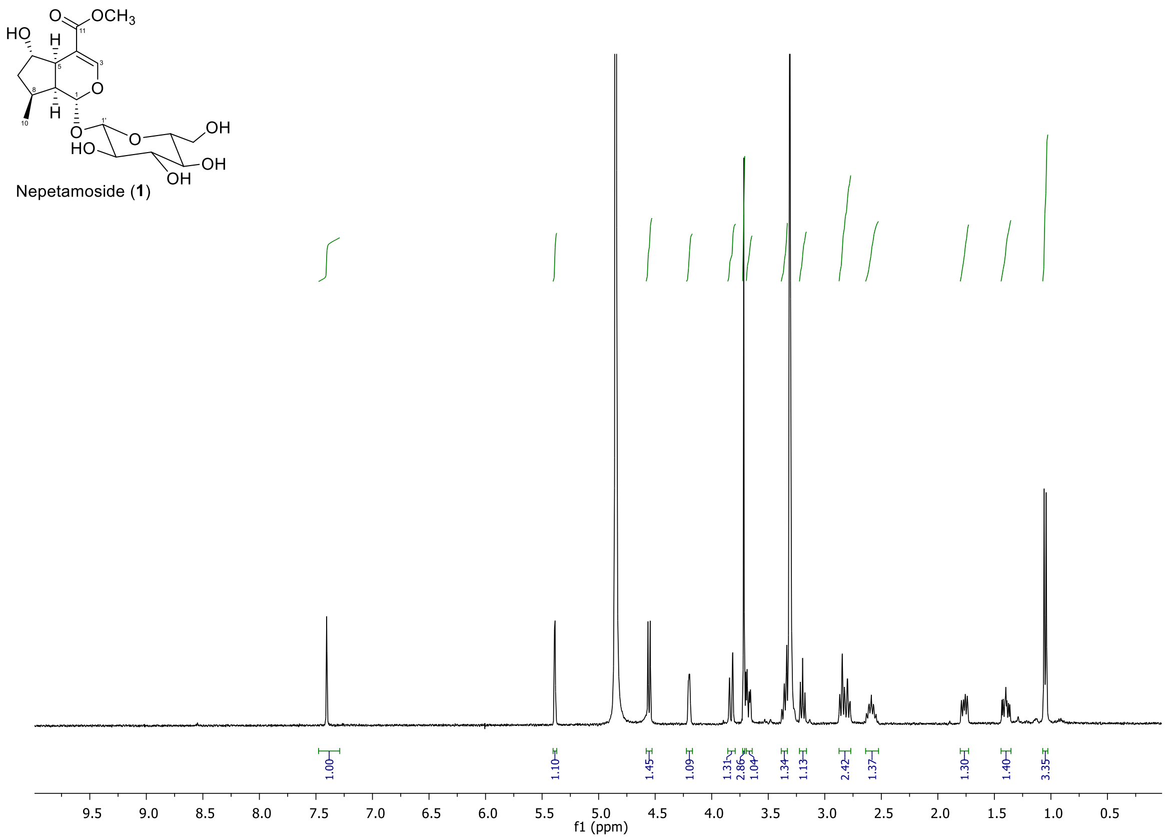

Figure S2. ^{13}C NMR (100 MHz, CD_3OD) of compound **1**

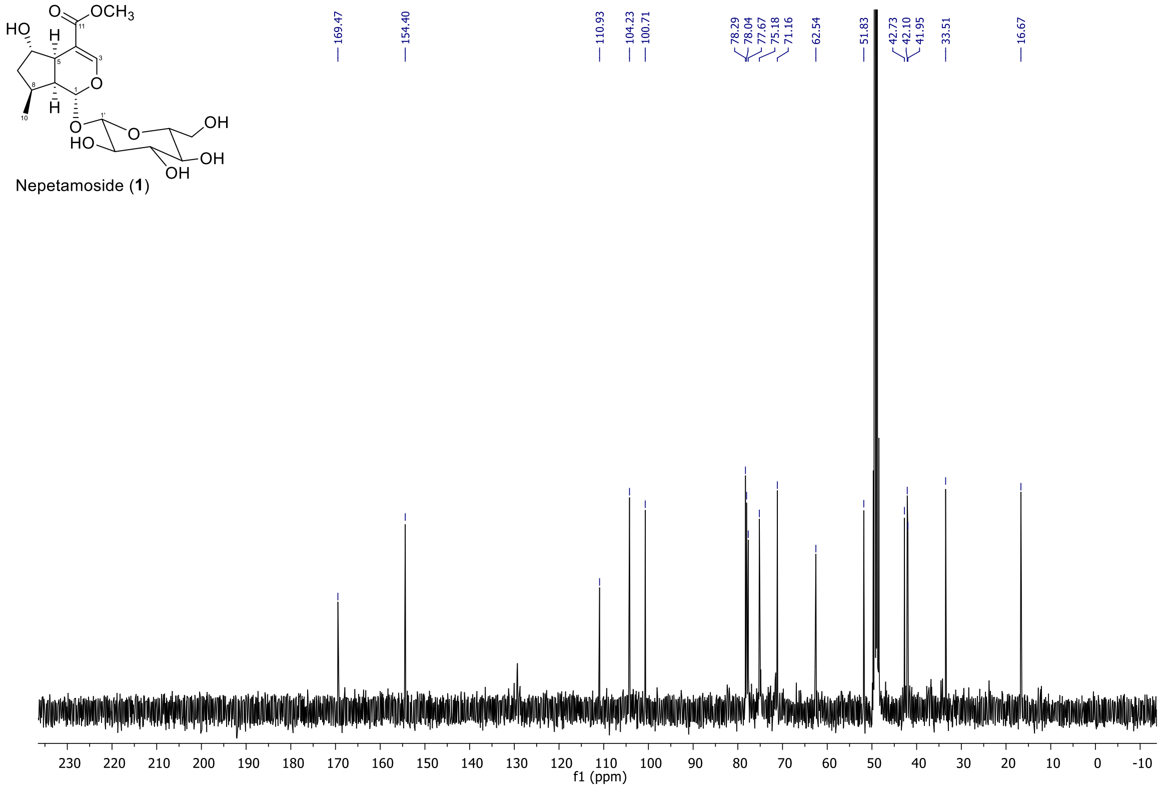

Figure S3. COSY spectrum (400 MHz, CD_3OD) of compound **1**

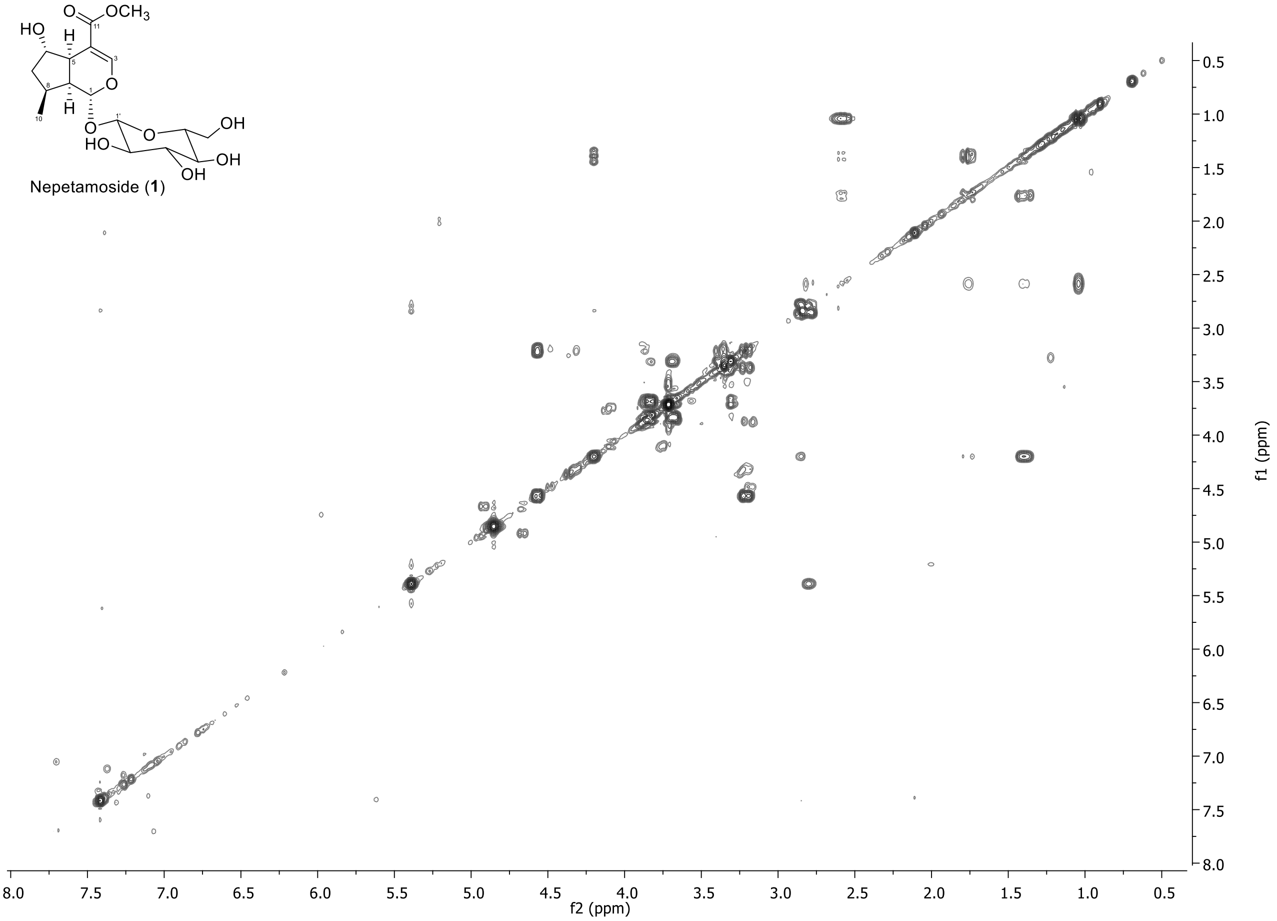

Figure S4. HSQC spectrum (400 MHz, CD_3OD) of compound **1**

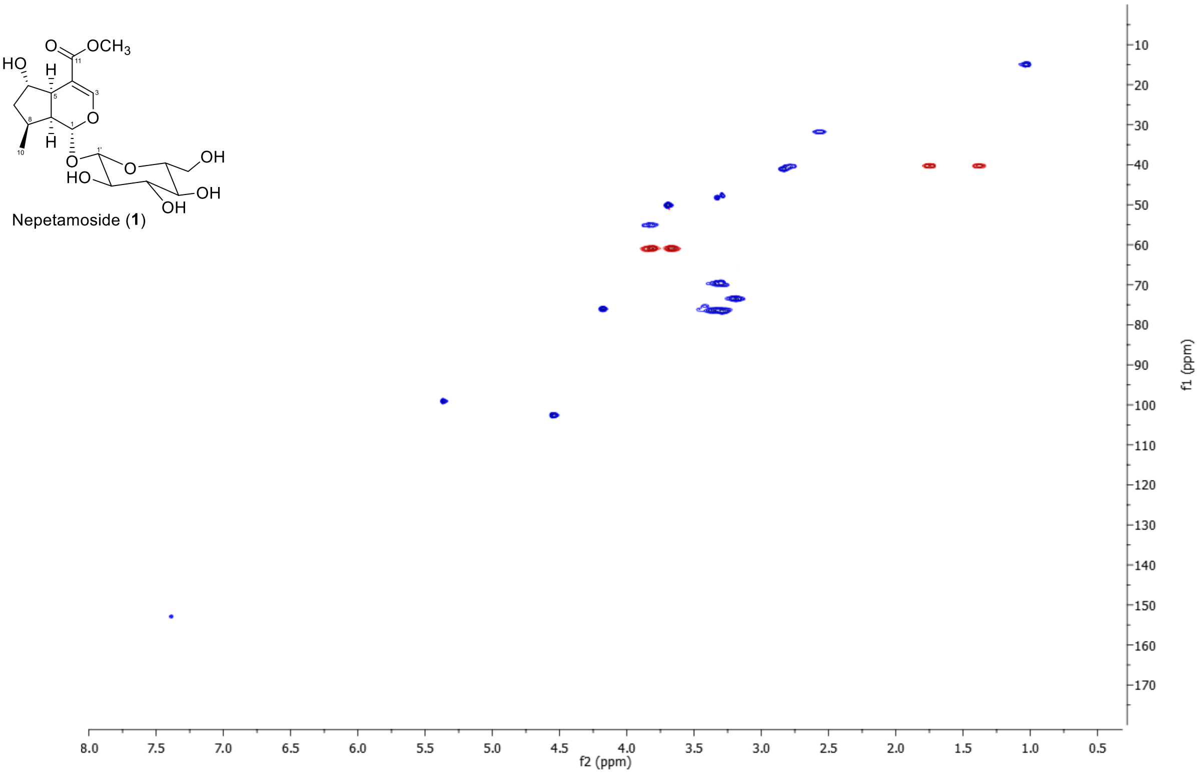

Figure S5. HMBC spectrum (400 MHz, CD_3OD) of compound **1**

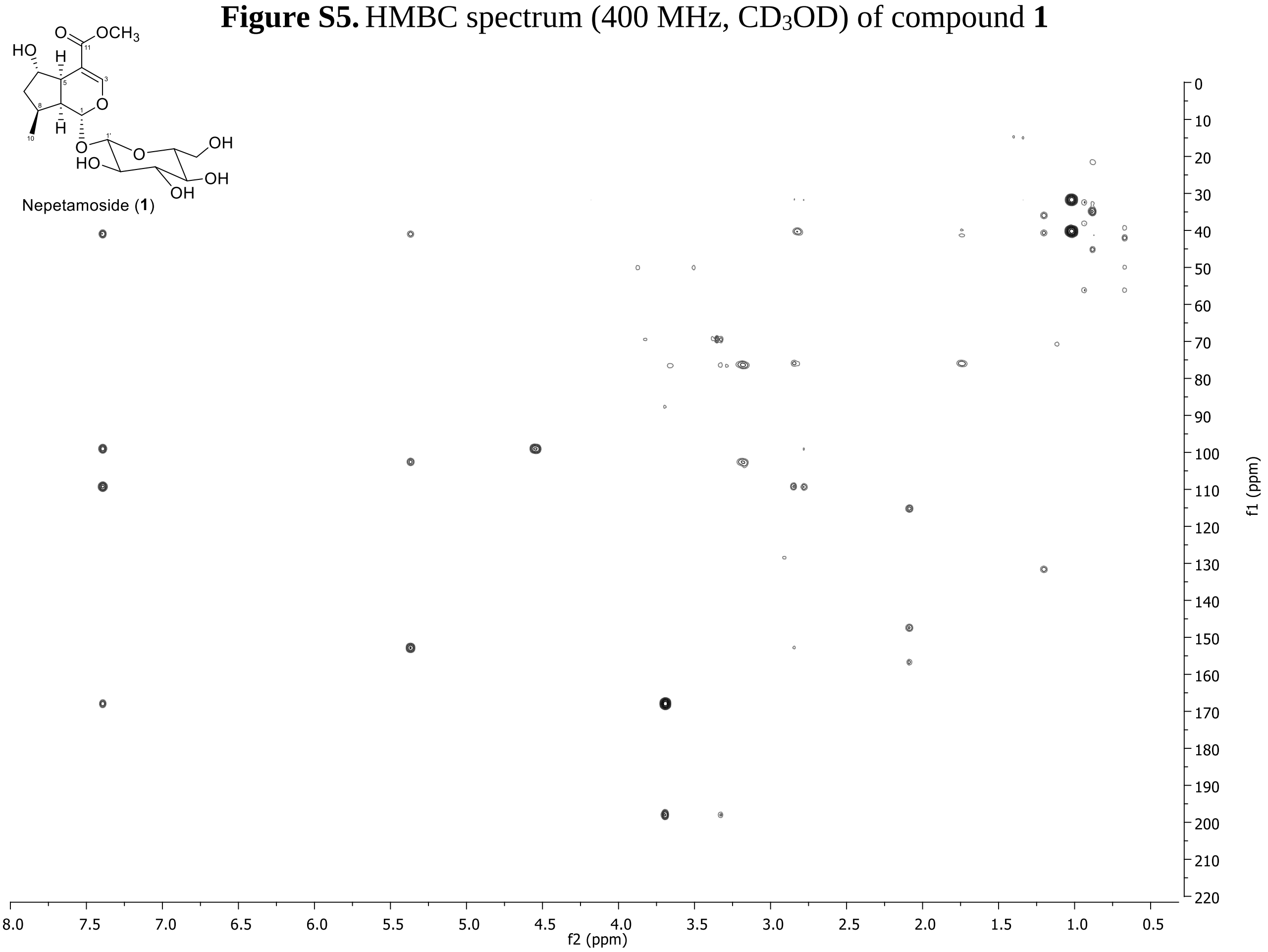

Figure S6. HRESIMS (positive ion mode) of compound **1**

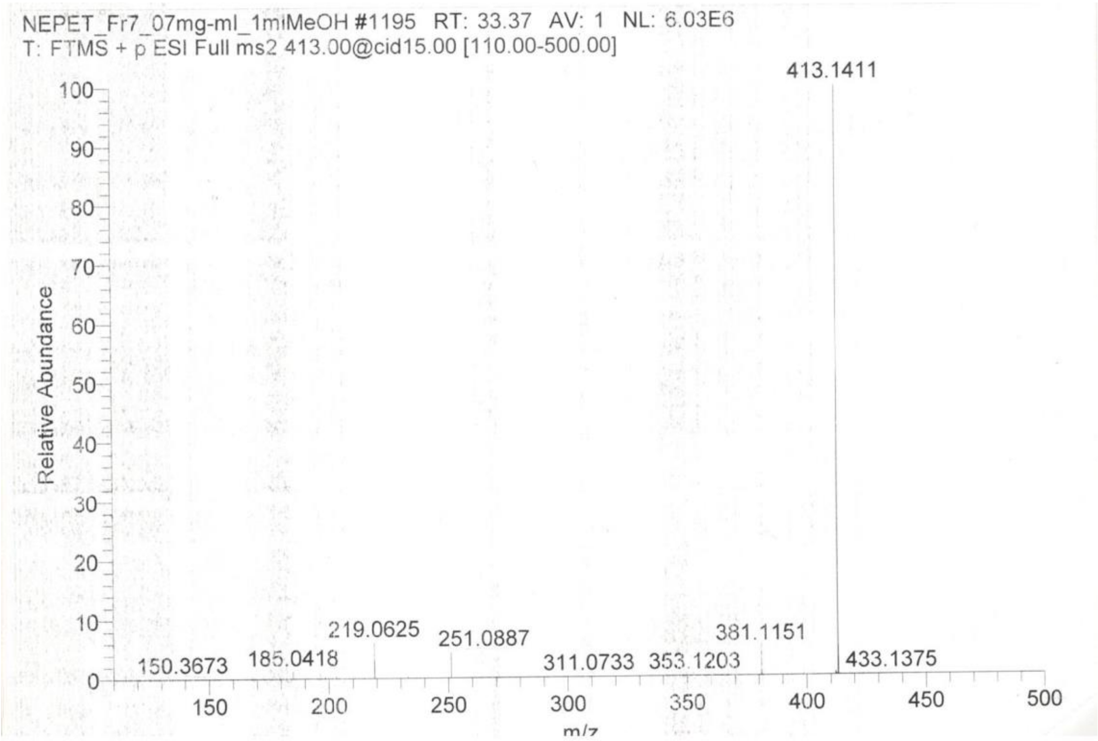

Figure S7. ^{1}H NMR (500 MHz, CD_3OD) of compound **2**

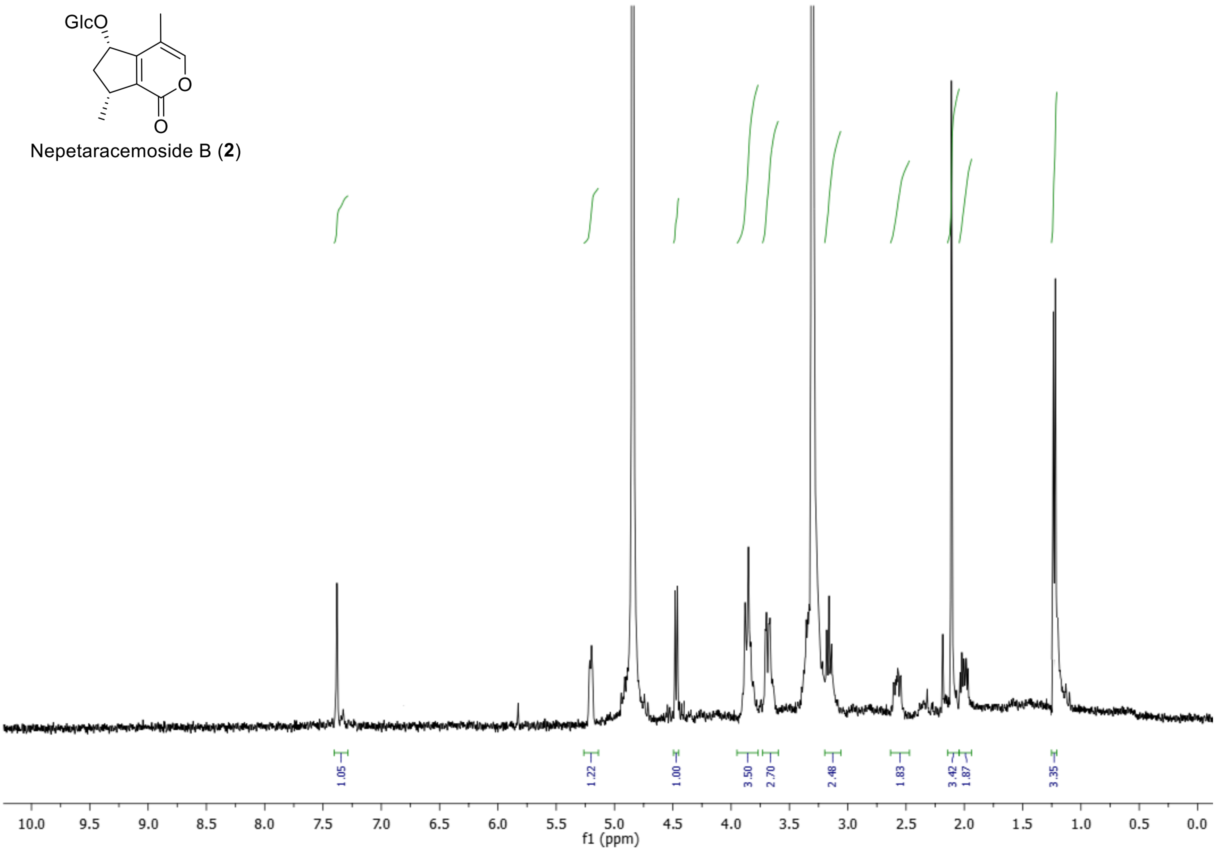

Figure S8. ^{1}H NMR (500 MHz, CD_3OD) of compound **3**

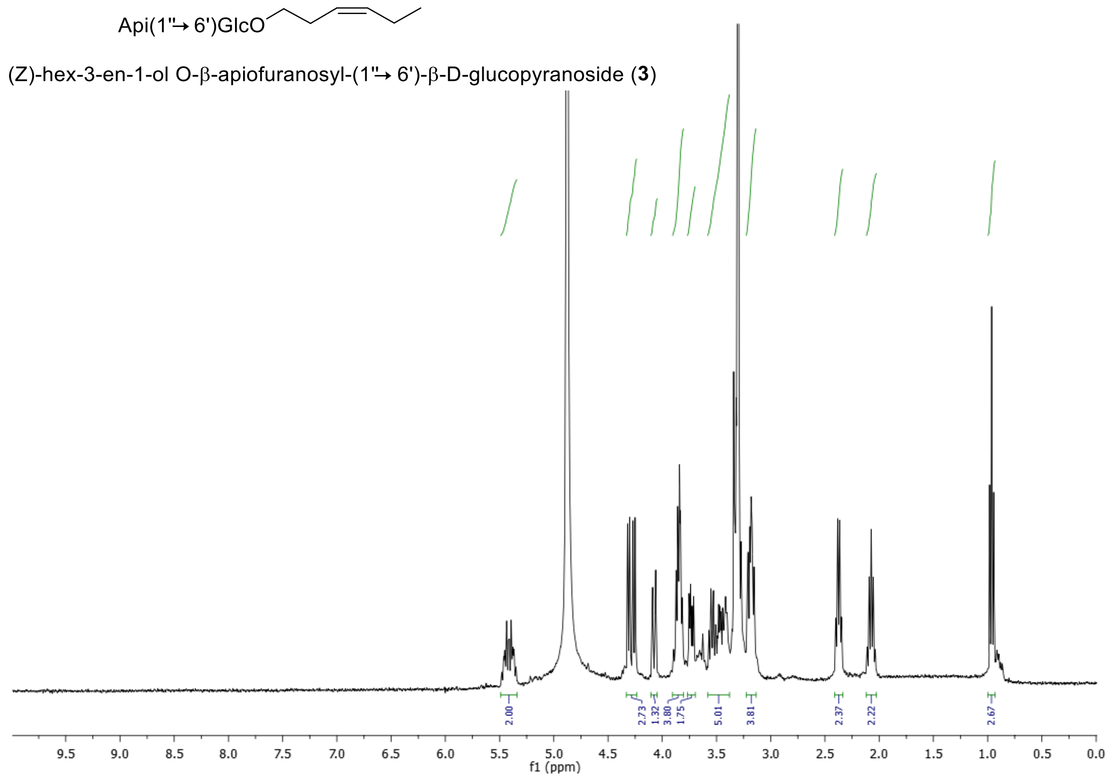

Figure S9. ^{1}H NMR (500 MHz, CD_3OD) of compound **4**

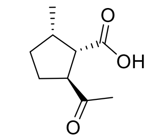

Nepetonic acid (**4**)

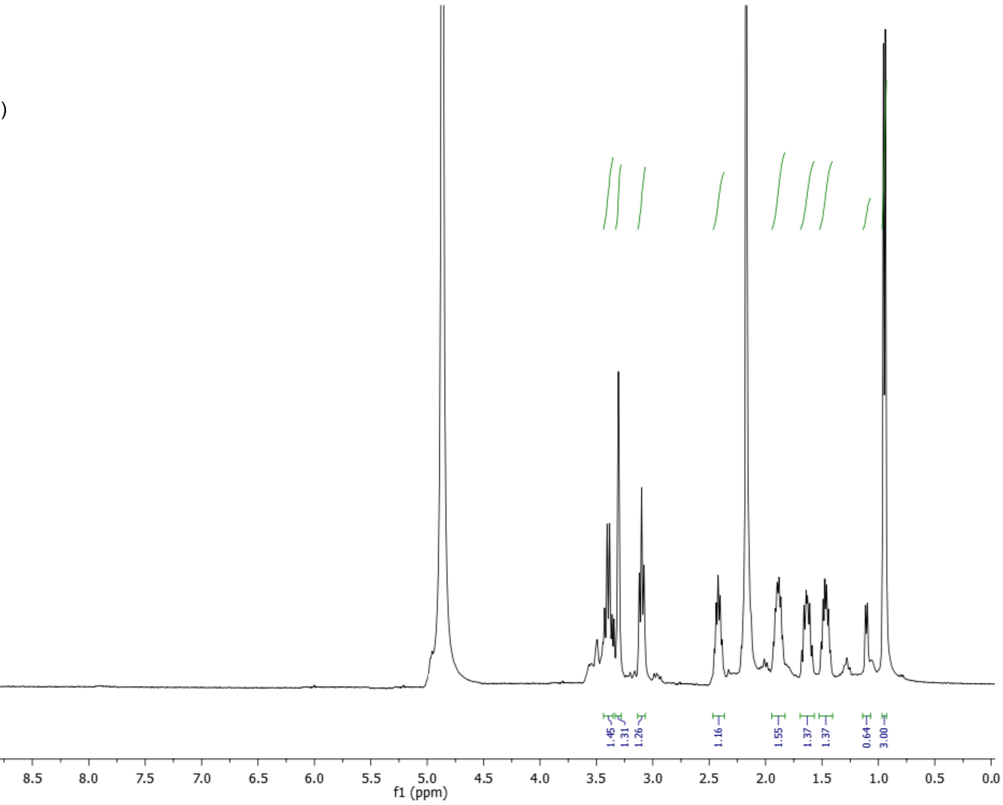

Figure S10. ^{1}H NMR (500 MHz, CD_3OD) of compound **5**

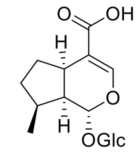

1,5,9-epideoxyloganic acid (**5**)

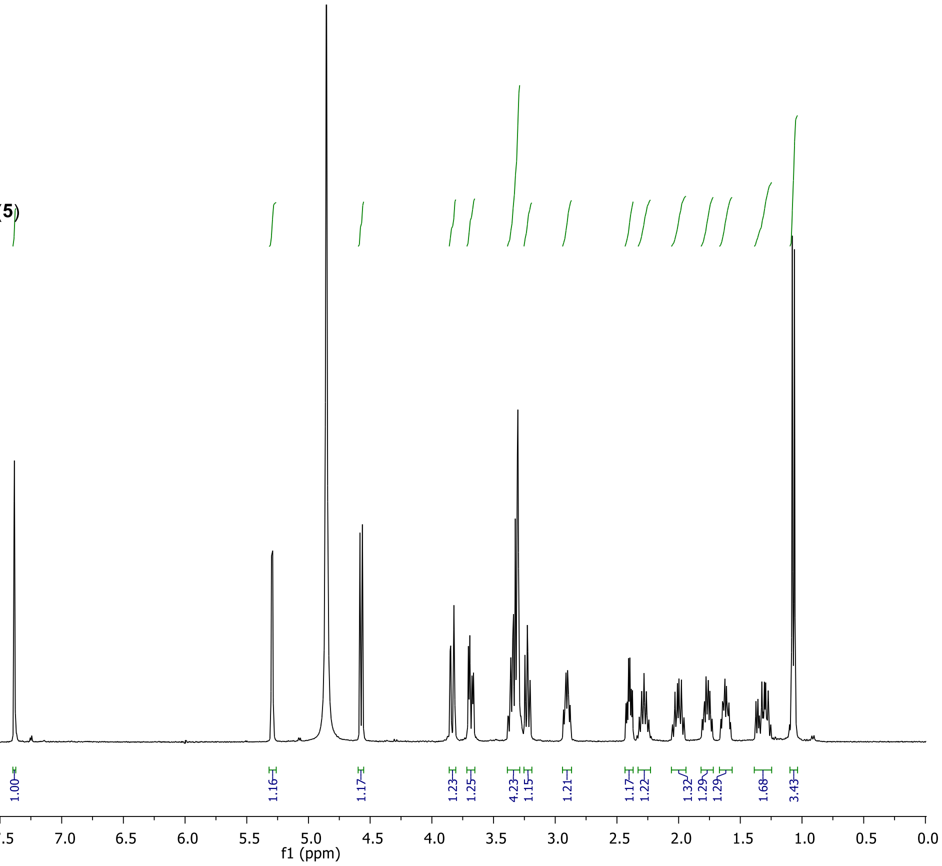

Figure S11. ^{1}H NMR (500 MHz, CD_3OD) of compound **6**

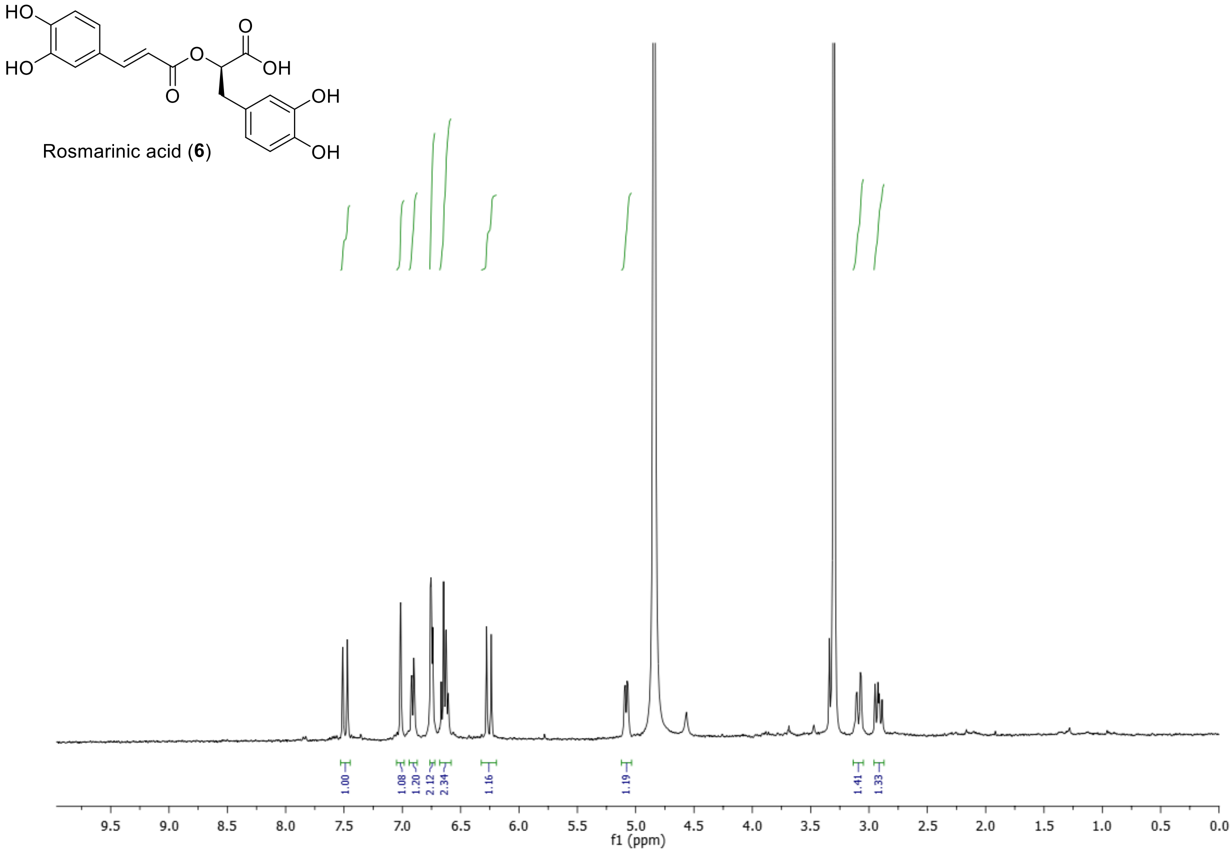

Figure S12. ^{1}H NMR (500 MHz, CD_3OD) of compound **7**

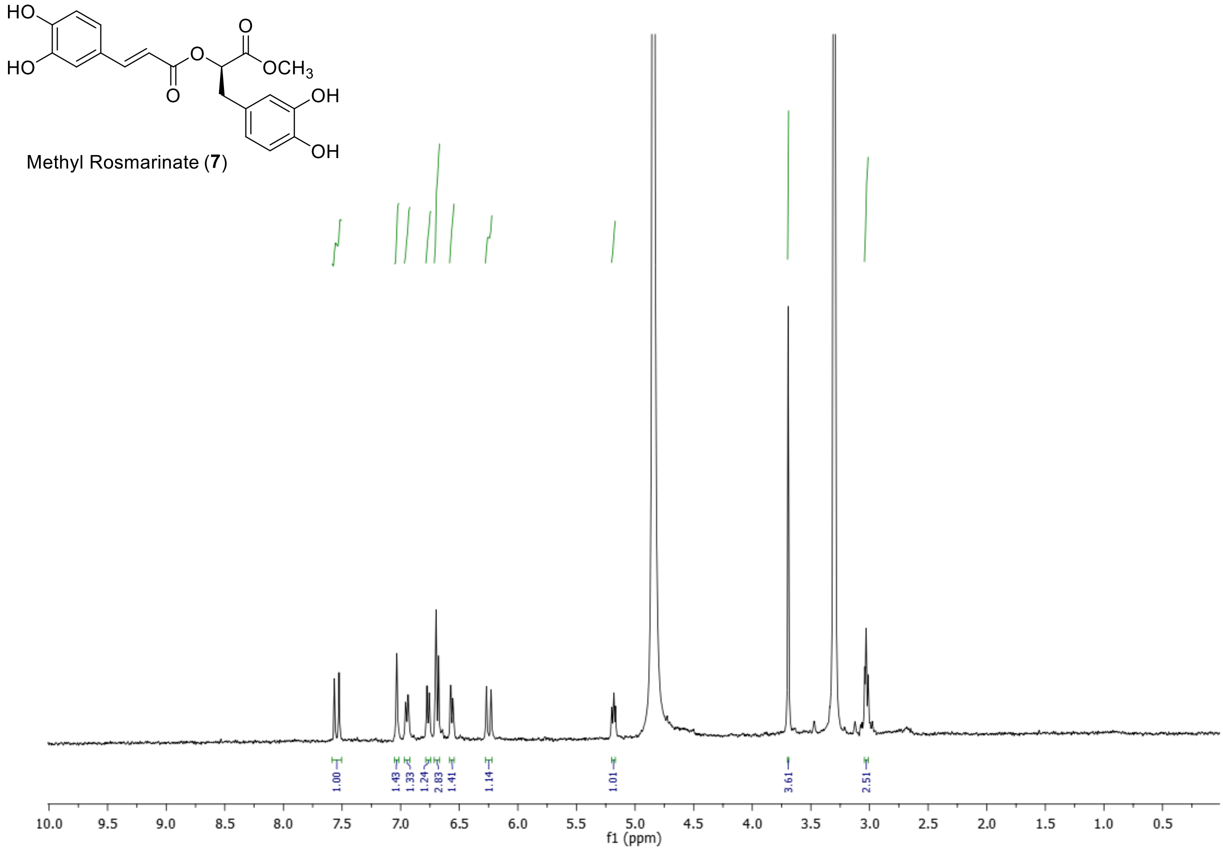

Figure S13. ^{1}H NMR (500 MHz, CD_3OD) of compound **8**

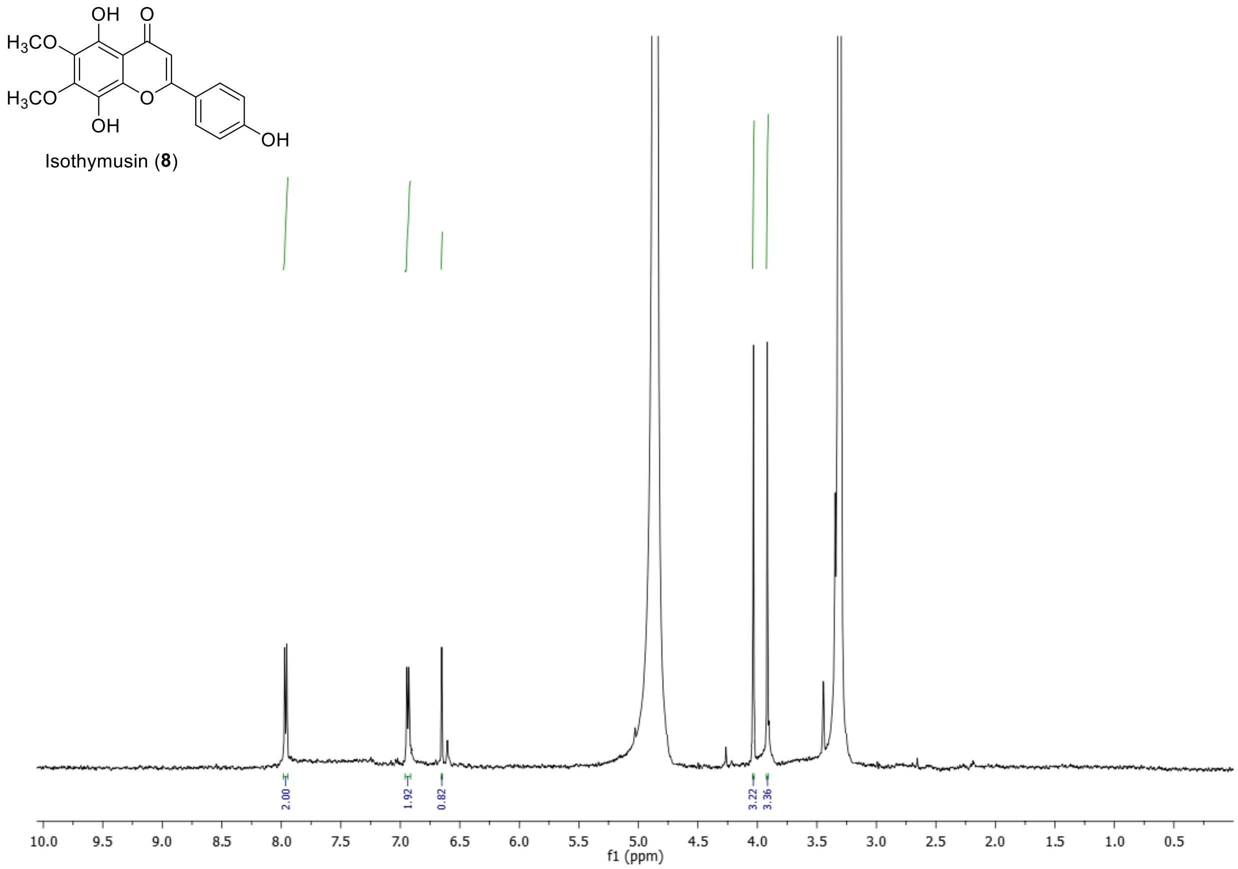

Figure S14. ^{1}H NMR (500 MHz, CD_3OD) of compound **9**

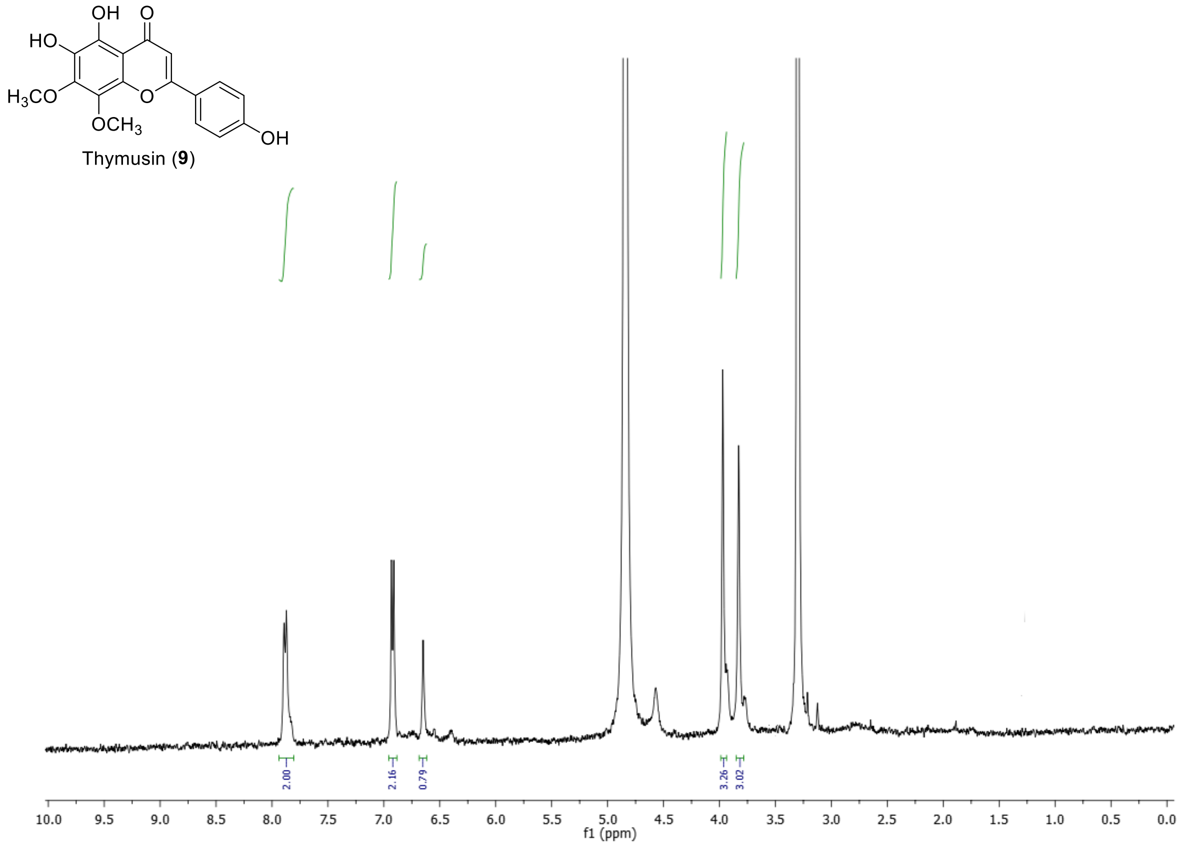

Figure S15. ^{1}H NMR (500 MHz, CD_3OD) of compound **10**

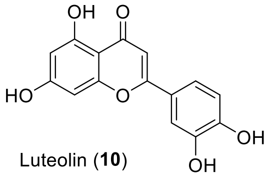

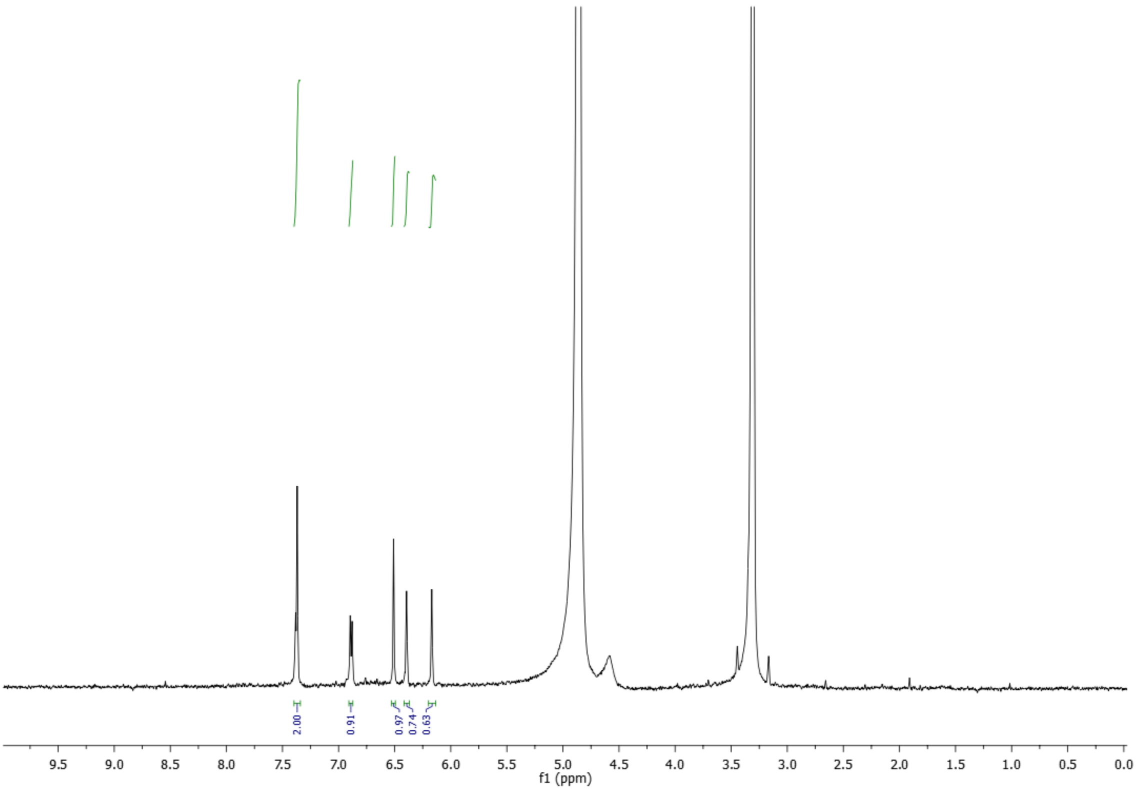

Figure S16. ^{1}H NMR (500 MHz, CD_3OD) of compound **11**

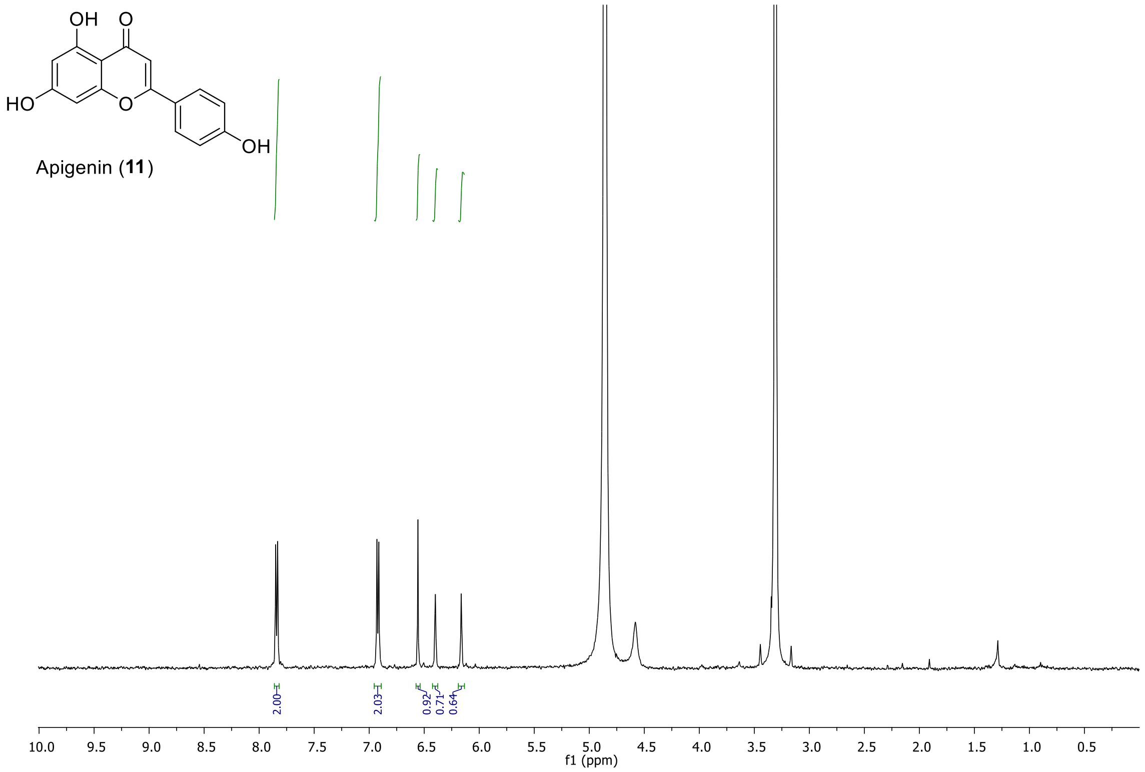

Supplement: Supplementary file 1 [file molecules-24-01684-s001.pdf]
